# Factors Associated with Response to Acetylcholinesterase Inhibition in Dementia: A Cohort Study from a Secondary Mental Health Care Case Register in London

**DOI:** 10.1371/journal.pone.0109484

**Published:** 2014-11-20

**Authors:** Gayan Perera, Mizanur Khondoker, Matthew Broadbent, Gerome Breen, Robert Stewart

**Affiliations:** 1 King's College London (Institute of Psychiatry), London, United Kingdom; 2 South London and Maudsley NHS Foundation Trust, London, United Kingdom; Alexander Fleming Biomedical Sciences Research Center, Greece

## Abstract

**Background:**

Acetylcholinesterase inhibitors (AChEIs) are widely used to delay cognitive decline in Alzheimer's disease. Observational studies in routine clinical practice have shown cognitive improvement in some groups of patients receiving these agents but longitudinal trajectories before and after AChEI initiation have not previously been considered.

**Objectives:**

To compare trajectories of cognitive function before and after AChEI initiation and investigate predictors of these differences.

**Method:**

A retrospective longitudinal study was constructed using data from 2460 patients who received AChEIs and who had routine data on cognitive function (Mini-Mental State Examination; MMSE) before and after AChEI initiation. Longitudinal MMSE change was modelled using three-piece linear mixed models with the following segments: 0–12 months prior to AChEI initiation, 0–6 months and 6–36 months after initiation.

**Results:**

MMSE decline was reversed (in that the slope was improved by an average 4.2 units per year, 95% CI 3.5–4.8) during the 6-month period following AChEI initiation compared with the slope in the one year period before AChEI initiation. The slope in the period from 6–36 months following AChEI initiation returned to the pre-initiation downward trajectory. The differences in slopes in the 1 year period prior to AChEI initiation and in the 6 months after initiation were smaller among those with higher MMSE scores at the time of AChEI initiation, among those who received a vascular dementia diagnosis at any point, and among those receiving antipsychotic agents.

**Conclusion:**

In this naturalistic observational study, changes in cognitive trajectories around AChEI initiation were similar to those reported in randomised controlled trials. The magnitude of the difference in slopes between the 1 year period prior to AChEI initiation and the 6 month period after AChEI initiation was related to level of cognitive function at treatment initiation, vascular comorbidity and antipsychotic use.

## Introduction

Dementia is a major challenge because of its increasing prevalence, substantial disability and high cost. Acetyl cholinesterase inhibitors (AChEIs) remain the mainstay of pharmacotherapy whose wide clinical use is largely justified by 3- to 6-month improvements in cognitive and global function in Alzheimer's disease with a Cochrane review estimating a 1.37 (95% CI 1.13, 1.61) Mini-Mental State Examination (MMSE) score improvement at 6 months after AChEI initiation [1]. Randomised controlled trials are optimally designed to quantify efficacy and/or effectiveness of interventions but tend to have insufficient power, even when combined, to describe individual variability in response, even less so predictors of good or poor response. Furthermore, trial inclusion and exclusion criteria limit generalisability of findings to ‘real world’ clinical settings. Findings from observational clinical studies of AChEI response have varied including an MMSE improvements of 0.5 points [2] and a 0.5 point decrease [3] at 6 months after treatment initiation. However, observational cohort studies to date have only investigated cognitive response to AChEIs from the point of drug initiation and have not taken into account the preceding trajectory of cognitive decline.

This study made use of a routine clinical data resource sourced from a large provider of dementia care, describing and comparing trajectories in MMSE scores before and after AChEI initiation followed by an investigation of factors associated with these changes in slope.

## Methods

### Ethics Statement

The study was approved as an anonymized dataset for secondary analysis by the Oxfordshire Research Ethics Committee C (reference 08/H0606/71).

### Study setting and data source

A retrospective observational study was conducted using data from the South London and Maudsley NHS Foundation Trust (SLAM) Biomedical Research Centre (BRC) Case Register. This data resource has been described in detail [4] and has supported a range of analyses [5], [6]. In summary, it provides researcher access to full but anonymised copies of electronic medical records from SLAM, Europe's largest mental healthcare provider covering a geographic catchment of 1.2 million residents and delivering a comprehensive range of mental health services, including dementia assessment and treatment. Data are currently archived on over 220,000 cases with a range of mental disorders.

### Study cohort

Using keyword searching functionality in the SLAM BRC Case Register, records were retrieved on all patients who had received AChEI drugs (donepezil, rivastigmine or galantamine) during 2003–2010 and information was manually retrieved including all recorded measures of cognitive function within 12 months before and 36 months after AChEI initiation. In UK clinical practice, AChEI agents were licensed for the treatment of Alzheimer's disease during the period of observation; however, in routine clinical practice there was flexibility in interpretation of this diagnosis – for example, in relation to comorbidity with vascular dementia. The sample for this analysis was of all people receiving these agents and recorded diagnoses were not applied as inclusion/exclusion criteria. In British dementia treatment services, cognitive function continues to be primarily monitored in routine clinical practice using the Mini-Mental State Examination (MMSE) [7] a 30-point measure of global function in wide use. In order to assess cognitive decline before and after AChEI initiation, the analysed sample was restricted to those with at least one MMSE score before AChEI initiation and one score afterwards. Date of AChEI initiation was defined as the first receipt of any of the three agents, regardless of dose.

### Measurements

All MMSE scores during the 4-year observation period were used to model cognitive trajectories. Entries describing scores with below-30 denominators due to missing items were corrected by the number of missing items. For example, if a 28 denominator was cited, 2 points were added to the numerator for analysis. In addition, the MMSE score closest to the date of AChEI initiation was categorised as a covariate into standard groups (25+, 21–24, 10–20, <10) for stratified analyses. The following additional information was extracted from the record using both structured fields and manual text review: age (at AChEI initiation), sex, ethnicity (white vs. other), and marital status (married/cohabiting vs. other). Highest educational qualification was categorised into three groups: i) no qualification, ii) standard qualification (O-level, implying school leaving age of 16), iii) advanced qualification (A-level or university, implying school leaving age above 16). As a proxy measure of socio-economic status, area-level deprivation scores in 2007 were available through an anonymised link created between lower super output area residence code (an address unit representing between 1500–2000 residents) and summary data for that area from 2001 UK Census output. The index of multiple deprivation is derived from seven domains: income, employment, health, education, housing and services, crime, and environment (Index of Multiple Deprivation, 2007). Patients not infrequently received more than one diagnosis during the course of their treatment and the presence of Alzheimer's disease, vascular dementia and Lewy body dementia diagnoses were ascertained as non-exclusive binary variables. The presence or not of a vascular dementia diagnosis at any point was considered as a proxy measure of comorbid cerebrovascular disease. Recorded medication was categorised into the following groups: antidepressants, antipsychotics, diabetes drugs, gastrointestinal drugs, lipid regulators, cardiovascular disease drugs, other central nervous system drugs, respiratory drugs, and antiplatelet, fibrinolytic or anticoagulant drugs.

### Statistical analysis

The analysed cohort was initially compared to the wider cohort of all patients receiving AChEI treatment. The trajectory of MMSE scores in the sample was initially modelled for observational purposes using a non-parametric regression method: the Generalised Additive Model for Location, Scale and Shape (GAMLSS) introduced by Rigby and Stasinopoulos [8]. This study used at least one single point of MMSE before and at least one MMSE point after drug initiation. After visual observation of this curve, a parametric piecewise linear mixed model, equivalent to a linear regression with interaction terms was used. For analysis the observation time was divided into the following three segments within which MMSE slopes (rate of change in MMSE scores) satisfactorily approximated linearity: i) 12 months prior to AChEI initiation; ii) 0 to 6 months after initiation; iii) 6 to 36 months after initiation. Slope differences were calculated between each of the three time segments (12 months prior, 0–6 months after and 6–36 after drug initiation). Time in the mixed models was defined as the number of years since the AChEI initiation date, with negative values for the pre-initiation period. Two-way interaction terms were used in the linear mixed estimations to estimate slope differences between time periods and were modelled before and after (Slope difference =  coefficient of time* before-after indicator). Three-way interaction terms were tested to investigate modification of slope differences by covariates (Modification of slope difference  =  Coefficient of time*before-after indicator*covariate). All analyses were carried out using R software.

## Results

Within the years of data examined, a total of 3416 patients had received AChEI treatment of whom 2460 had sufficient MMSE scores for analysis. Characteristics of these samples are described and compared in Table 1; in summary, there were no observable differences and the analysed sample appeared representative. For the analysed sample within the observation period, data on a total of 10,669 MMSE scores were available, with a mean 4.3 (SD 0.51) scores per patient. The mean MMSE score at AChEI initiation was 19.9 (SD 5.1).

**Table 1:** Comparison of all patients received AChEI treatment (full cohort) and the analysed sample.

Characteristic	Full cohort (n = 3416)	Analysed sample* (n = 2460)
MMSE score at drug initiation		
*25 and over*	705 (20.6%)	529 (21.5%)
*21–24*	913 (26.7%)	706 (28.7%)
*20–10*	1633 (47.8%)	1161 (47.2%)
*less than 10*	165 (4.8%)	64 (2.6%)
Age at AChEI initiation		
*<65 years*	123 (3.6%)	89 (3.6%)
*65–79 years*	1390 (40.7%)	1028 (41.8%)
*80+ years*	1903 (55.7%)	1343 (54.6%)
Gender		
*Male*	1208 (35.4%)	870 (35.4%)
*Female*	2207 (64.6%)	1590 (64.6%)
Ethnicity		
*White*	2808 (83.8%)	2044 (84.1%)
*Non-white*	543 (16.2%)	386 (15.9%)
Marital status		
*Married/cohabiting*	1332 (41.4%)	953 (40.4%)
*Divorced/single/separated or widowed*	1885 (58.6%)	1404 (59.6%)
Education		
*No qualifications*	784 (33.4%)	630 (35.1%)
*Standard qualification*	451 (19.2%)	352 (19.6%)
*Advanced qualification*	1114 (47.5%)	811 (45.2%)
Deprivation quintile		
*1 (most deprived)*	741 (22.0%)	521 (21.5%)
*2*	689 (20.5%)	466 (19.2%)
*3*	638 (19.0%)	459 (18.9%)
*4*	889 (26.4%)	668 (27.5%)
*5 (least deprived)*	408 (12.1%)	314 (12.9%)
Type of dementia diagnosed at any point		
*Alzheimer's disease*	2760 (80.8%)	2050 (83.3%)
*Vascular dementia*	673 (19.7%)	491 (19.9%)
*Lewy body dementia*	118 (3.4%)	89 (1.8%)

*Sufficient MMSE data (i.e. at least one score within 12 months before and one score within 36 months after treatment initiation) and measurement restricted to 12 months before and 36 months after treatment initiation.

**Figure 1:**
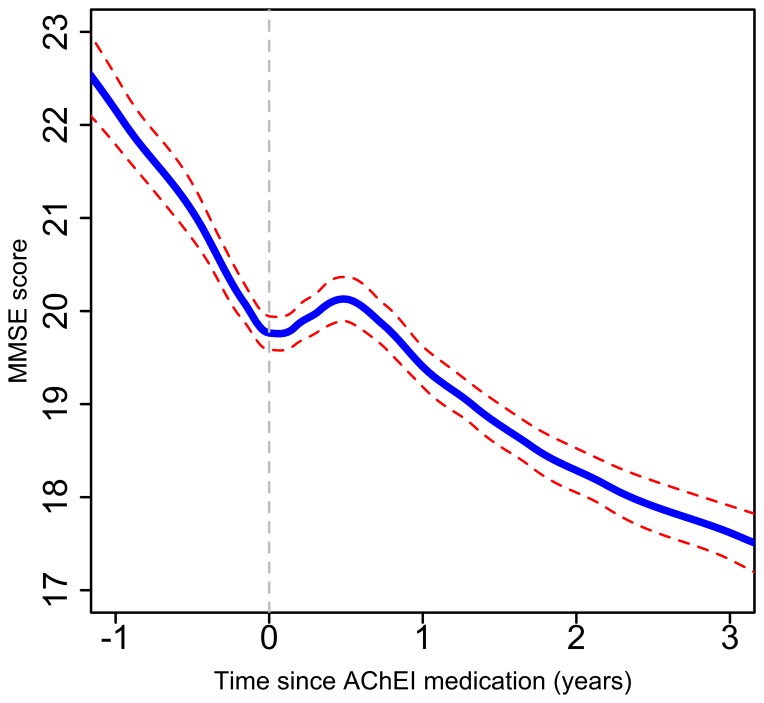
Non-parametric (GAMLSS) models of MMSE trajectories before and after AChEI initiation for all patients who received AChEIs (n = 2460), (AChEI initiation date is defined as time 0).

Figure 1 displays non-parametric (GAMLSS) models of MMSE slope trajectories before and after AChEI initiation for the whole sample and stratified by MMSE strata at treatment initiation. It was observed that a pre-treatment decline was followed by an improvement over the 6 months after treatment, which in turn was followed by continued decline. For the full cohort, following the initial score improvement the MMSE at treatment initiation was reached again after 10 (95% CI 7–12) months. An additional observation was that the 6-month improvement became progressively more marked with lower MMSE at AChEI initiation.

From parametric (piecewise linear mixed) models, the three MMSE slopes were calculated to be a decline of −2.45 (95% CI −2.88, −2.02) points per year during the 12 months prior to AChEI initiation, an increase of 1.73 (1.34, 2.12) points per year during the 6 months after initiation, and a decline of −2.68 (−2.86, −2.50) points per year during the 6–36 month period. Differences in slopes for the whole sample and by MMSE strata are displayed in Table 2. For the whole sample significant slope improvements were observed over 0–6 months compared to those in the 12 months pre-treatment; however, no significant slope difference was found comparing the 6–36 month and pre-treatment periods. As had been suggested in non-parametric models, the 0–6 month slope improvement was substantially more marked in patients with lower MMSE scores at AChEI initiation. In those with lowest (below 10) scores, 6–36 month MMSE slopes were also improved compared to those in the pre-treatment period, although confidence intervals were wide.

**Table 2:** Difference in unadjusted and adjusted MMSE decline rate per year for different baseline MMSE scores.

		Unadjusted rate		Adjusted rate$	
Time periods compared	Baseline MMSE	Differences in slope, MMSE points per year	95% CI	Differences in slope, MMSE points per year	95% CI
	Total sample	4.19	3.52 to 4.85	4.16	3.49 to 4.83
0 to 6 months post-treatment vs. 12 months pre-treatment	25 and over	0.52	−0.66 to 1.70	0.49	−0.69 to 1.67
	21–24	2.96	2.02 to 3.91	2.96	2.37 to 3.55
	10–20	5.93	4.97 to 6.89	5.9	4.94 to 6.86
	<10	9.4	4.83 to 14.0	9.74	5.13 to 14.40
	Total sample	−0.22	−0.65 to 0.21	−0.23	−0.66 to 0.20
6 to 36 months post-treatment vs. 12 months pre-treatment	25 and over	−1.36	−2.09 to −0.63	−1.37	−2.10 to −0.64
	21–24	−0.81	−1.42 to −0.20	−0.80	−2.72 to 1.12
	10–20	0.09	−0.56 to 0.74	0.09	−0.56 to 0.74
	<10	3.37	0.27 to 6.47	3.59	0.45 to 0.73

$ adjusted for age at AChEI initiation, gender, marital status, education, ethnicity and deprivation.

Considering the 6-month MMSE slope improvement, further interactions with socio-demographic and clinical covariates are summarized in Table 3. No significant differences were found in slope improvement between comparison periods by age, gender, deprivation, education or marital status. However, improvement in MMSE slope after treatment initiation was significantly stronger in non-white ethnic groups. It was weaker in patients who had received a vascular dementia diagnosis, but no modification was found by presence or not of a Lewy body dementia diagnosis. The same interactions with co-administered medications are summarised in Table 4: use of antipsychotic agents, gastrointestinal drugs and anti-platelet, fibrinolytic or anticoagulant agents were all associated with a smaller slope difference between time periods. When analyses were repeated restricting medications to those recorded before AChEI initiation, the interaction terms for antipsychotics (coefficient = 0.16, P-value = 0.66) and gastrointestinal drugs (coefficient = 0.28, P-value = 0.54) were substantially reduced but the coefficient for anticoagulant drug use remained similar (coefficient was 0.58, p = 0.09).

**Table 3:** Socio-demographic/clinical characteristics and MMSE slope differences before and 6 months after AChEI initiation.

Predictor	Slope difference	Coefficient	Standard error	p-value
Unstratified (total sample)	4.16			
Age at AChEI drug initiation				
*65–69*	4.14	0.002 (per year increase)	0.01	0.71
*70–74*	4.15			
*75–79*	4.16			
*80–84*	4.17			
*85–89*	4.18			
*90+*	4.19			
Gender				
*Mal*e	4.10	Ref.		
*Female*	4.21	0.11	0.2	0.42
Ethnicity				
*White*	4.09	Ref		
*Non-white*	4.60	0.51	0.2	0.009
*Deprivation quintile*				
*1 (most deprived)*	4.20	0.01	0.01	0.53
*2*	4.22			
*3*	4.19			
*4*	4.14			
*5 (least deprived)*	4.09			
*Marital status*				
*Married/cohabiting*	3.98	Ref.		
*Not cohabiting*	4.28	0.3	0.1	0.08
*Education*				
*No qualification*	4.08	0.04	0.13	0.65
*O/Level*	4.13			
*A/level or University*	4.16	.		
Any vascular dementia diagnosis				
*Present*	3.80	−0.41	0.21	0.04
*Not present*	4.19	Ref.		
Any Lewy body dementia diagnosis				
Present	4.22	0.07	0.44	0.87
Not present	4.16	Ref.		

**Table 4:** Co-administered medication and MMSE slope differences before and 6 months after AChEI initiation.

Predictor	Slope difference	Coefficient	Standard error	p-value
Antidepressants				
*Received*	4.21	0.06	0.11	0.81
*Not received*	4.15	Ref.		
Antipsychotics				
*Received*	3.73	−0.73	0.25	0.008
*Not received*	4.46	Ref.		
Other CNS drugs				
*Received*	4.14	−0.22	0.12	0.21
*Not received*	4.36	Ref.		
Diabetes drugs				
*Received*	4.22	0.12	0.21	0.51
*Not received*	4.14	Ref.		
Gastrointestinal drugs				
*Received*	3.91	−0.34	0.12	0.006
*Not received*	4.25	Ref.		
Lipid lowering agents				
*Received*	4.37	0.35	0.24	0.13
*Not received*	4.12	Ref.		
CVD drugs				
*Received*	4.21	0.1	0.11	0.94
*Not received*	4.11			
Anti-platelet, fibrinolytic and anticoagulant agents				
*Received*	4.01	−0.33	0.12	0.02
*Not received*	4.34	Ref.		
Respiratory system drugs				
*Received*	4.33	0.22	0.51	0.67
*Not received*	4.11	Ref.		

## Discussion

**Figure 2:**
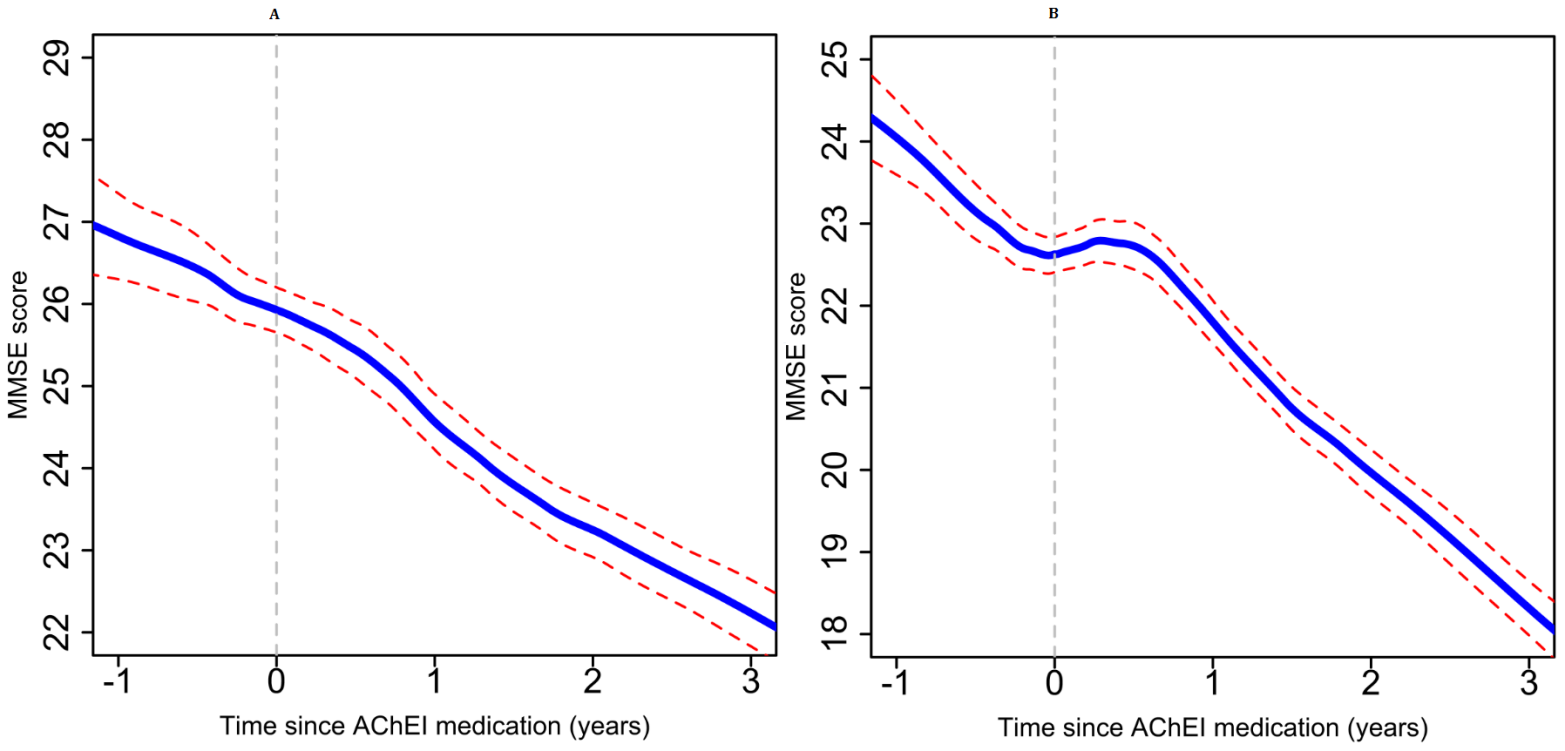
Non-parametric (GAMLSS) models of MMSE trajectories before and after AChEI initiation for those patients whose MMSE scores at the time of AChEI initiation were: (A) between 25 and 30, (B) between 21 and 24, (AChEI initiation date defined as time 0).

**Figure 3:**
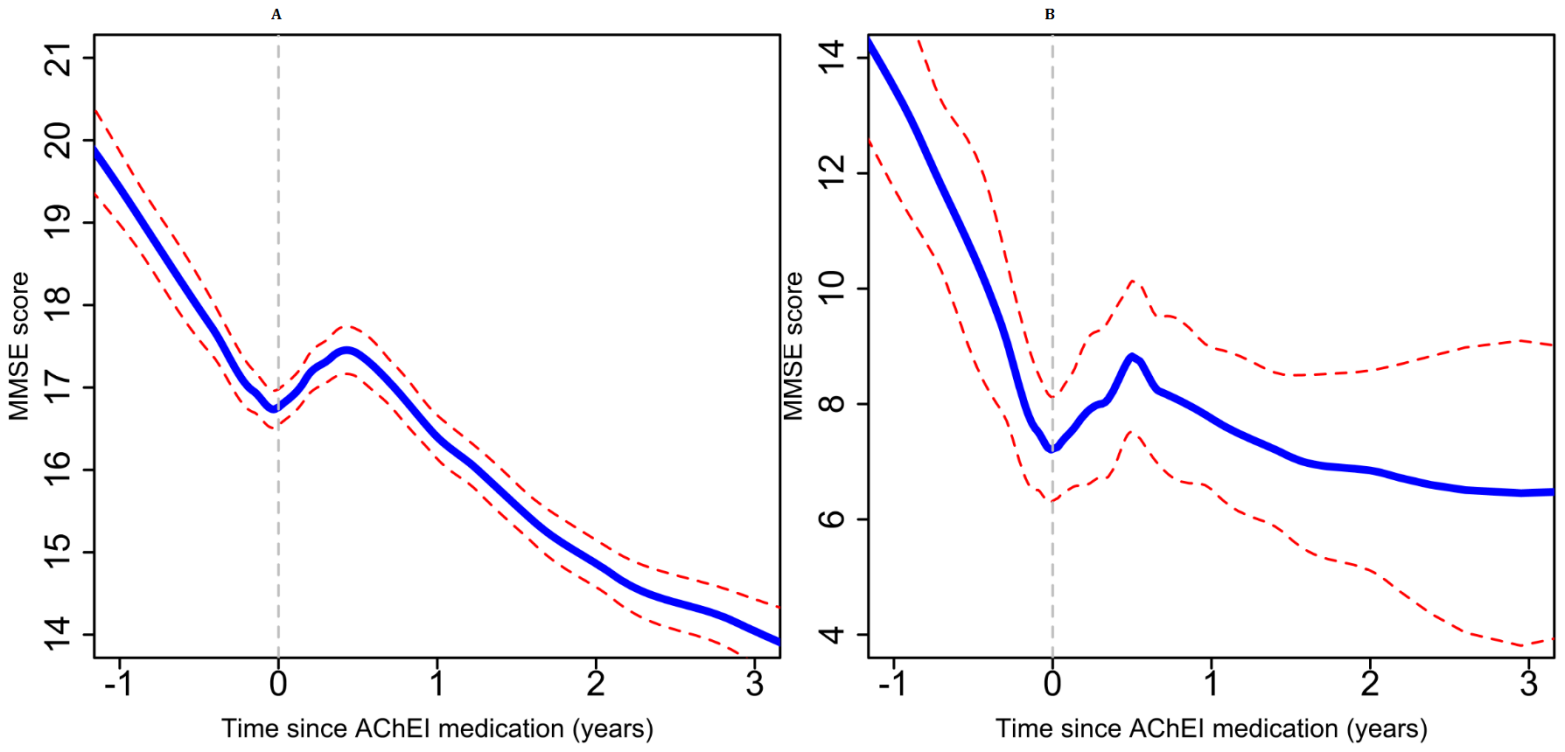
Non-parametric (GAMLSS) models of MMSE trajectories before and after AChEI initiation for those patients whose MMSE scores at the time of AChEI initiation were: (A) between 10 and 20, (B) between 0 and 10, (AChEI initiation date defined as time 0).

In this large observational study of routine AChEI treatment provision, we investigated the difference between pre- and post-treatment trajectories of cognitive function and predictors of these slope differences. On average, a significant improvement was observed in the MMSE trajectory by 4.16 points per year in patients who received AChEIs during the 6 months after treatment compared with the 12 months before treatment. However, MMSE slopes after 6 months were near-identical to pre-treatment slopes in most cases. When stratified by MMSE score at AChEI initiation, people with higher scores (MMSE scores 21 or over) at the time of AChEI initiation on average had a smaller improvement (figure 2) than people with lower scores (MMSE score <21) at the time of AChEI initiation (figure 3). Most socio-demographic factors investigated were not predictors of the initial slope change, including age at treatment initiation, gender, and education. However, non-white ethnic groups who received AChEI treatment responded significantly better, with a slope improvement more marked than their counterparts by 0.5 points per year. In terms of diagnosis, patients with vascular dementia recorded at any point responded less well (by 0.4 MMSE points per year) than those without this diagnosis. Patients who received antipsychotics, gastrointestinal drugs, and anti-platelet, fibrinolytic or anticoagulant agents at any point were found to have a weaker response to AChEIs, although only the last of these persisted as an effect modifier when pharmacotherapy was restricted to that recorded before AChEI treatment initiation.

Randomised controlled trials of AChEI treatment in Alzheimer's disease specifically have demonstrated an improvement in MMSE score in the intervention arm at 6 months. In our naturalistic sample from routine clinical practice, the pattern was interestingly similar with a slope of 1.73 MMSE points per year improvement over the 6 months after treatment initiation. As previously stated, observational studies to date have only described slopes following treatment, but have given rise to heterogeneous findings. One UK study found an improvement of 1.8 MMSE points per year among 939 patients with a mean follow up period of 4 months [9]. However, another study found no change over 6 months in people receiving donepezil [10], a finding that concurred with other studies of donepezil in clinical practice over the same period [11], suggesting stabilisation of cognitive decline rather than improvement over the first 6 months. A Spanish clinical cohort found over a 6 month period that the MMSE score improved significantly over baseline [12], but an Italian study found a one point per year decline for all three AChEI agents, although the slope did not show a statistically significant difference from a zero slope [3].

Several studies have suggested that MMSE score at baseline is a predictor of response to AChEIs [13], although a Cochrane review concluded that all three agents were efficacious for mild to moderate Alzheimer's disease [1]. Studies have also shown that as AD progresses, AChE activity can be reduced by up to 67% of normal levels in the temporal lobe and hippocampus [14]. An observational study found a better cognitive response to AChEI treatment in patients with moderate dementia (MMSE 10–20) compared to those with mild dementia (MMSE 21 or over) at baseline [9]. At least part of the difference in apparent response may relate to the underlying rate of cognitive decline at different stages of dementia, with more detectable effects at later stages in the context of accelerated progression [9]. The finding that those with moderate dementia do better, in terms of cognitive improvement, than those with mild dementia replicates the findings of other studies of routine clinical populations [15]. This might be explained by differences in the meaning of a point difference in MMSE across its distribution: i.e. a difference may be more readily detected at moderate than mild dementia ranges. Furthermore, it is possible that there is a greater reversible cholinergic deficit in patients with moderate dementia [9]. Post-mortem studies in AD have shown measures of cholinergic function to correlate with global cognitive test scores [16], and AChEIs may have a greater effect on a larger cholinergic deficit [9].

Cognitive improvement following AChEI treatment was found to be stronger in patients from non-white compared to white ethnic groups. In the geographic catchment covered by SLAM, non-white elders are predominantly African-Caribbean, although it was not possible to break down non-white groupings into more specific subtypes. Higher prevalence of dementia has been found in older African- Caribbean compared to white UK-born residents as well as a dementia onset in African-Caribbean patients nearly, on average, 8 years younger than their white counterparts [17]. Vascular dementia is likely to be a more common feature in African-Caribbean elders because of their well-recognised higher risk of stroke, hypertension and diabetes. However, neither vascular disease nor a younger age of onset are likely to account for the better response to AChEI treatment because, in the sample as a whole, age of onset was not associated with treatment response and vascular dementia was associated with a less marked response. This finding therefore requires replication and further investigation. One possible explanation might lie in more supportive family structures and consequent improved treatment adherence.

Considering dementia diagnoses as covariates, the presence of vascular dementia was associated with a significantly weaker difference between pre- and post-treatment slopes. Previous longitudinal studies in dementia have found accelerated cognitive decline associated with cerebral infarcts and congophilic angiopathy [18], and slower progression associated with diabetes, but no associations with hypertension and hypercholesterolemia [19]. However, these findings for cognitive decline in dementia do not necessarily predict treatment response. Diagnoses recorded were those applied in routine practice by clinicians and should not be assumed to generalise to research diagnoses. However, the finding of a lower AChEI response in cases receiving a vascular dementia diagnosis may reflect a weaker cholinergic deficit in this group [20], or might possibly reflect a diagnosis made or altered on the basis of worse treatment response (reverse causality). Although a diagnosis of vascular dementia was associated with a smaller response to AChEI treatment, there was only limited evidence for the same pattern of association with specific cardiovascular medication groups apart from anticoagulant and related agents, which were also associated with reduced likelihood of response. Since aspirin is likely to be the most common agent in this category, the effect modification may primarily relate to the presence of cerebrovascular disease rather than risk factors for this.

Analyses of other medications found that receipt of gastrointestinal drugs, anticoagulant agents, and antipsychotic agents was associated with smaller MMSE slope differences after AChEI initiation. In a pooled analysis of the placebo arms of six AD trials, antipsychotic use was found to be associated with significantly lower cognitive function [21] and Lopez- Pousa et al (2006)[12] found that patients treated with atypical antipsychotics had a greater risk of mortality, although as with other studies of this kind, it is difficult to conclude whether the mortality was a direct consequence of antipsychotic use or secondary to the symptom or behavioural profiles for which they were prescribed. Other studies have found an association between the use of atypical antipsychotic drugs and a greater frequency of cerebrovascular outcomes [22]. Although our observations might reflect adverse effects of antipsychotic agents on cognition accounting for the reduced AChEI response, this effect modification could not be demonstrated when restricted to exposure in the pre-AChEI period. Thus it was not possible to exclude the possibility that antipsychotic agents were being used to treat a more severe dementia syndrome which had failed to respond to AChEI treatment.

Our study found that a better improvement in MMSE score after AChEI treatment was present in patients of non-white ethnicity, those without vascular dementia diagnosis at any point, those not receiving antipsychotic agents, those not receiving gastrointestinal drugs, and those not receiving anti-platelet, fibrinolytic and anticoagulant agents. However, although these MMSE trajectory differences were statistically significant, they were relatively small in size and detectable as a group-level effect over a large sample. It cannot therefore be concluded from these data that they were sufficiently clinically significant to warrant alterations in routine practice – this would require a longer-term comparative evaluation of subsequent disease course and of broader outcomes beyond cognitive function, which was outside the scope of this study.

This study had considerable advantages over other comparable cohorts, not only in the data available on pre-treatment MMSE slopes but also in the substantial accumulated follow-up – approximately 5900 person years in total (1700 person years pre-treatment, 4200 post-treatment). Although case registers have a long history and are widely used in mental health and dementia research, we believe that the SLAM BRC Case Register is likely to be the largest in Europe at least containing the necessary depth of data for this type of study [4], other data resources tending to be confined to summary or administrative variables rather than full text records. The ‘before and after' comparison is a powerful means to limit confounding because the effect is inferred from pooling within-individual comparisons (i.e. are matched for the characteristics of the individual). The key potential confounding factors in such a comparison are other features which have also varied before and after treatment and are therefore substantially more limited.

The key limitations concern the observational nature of the study and the fact that data were from routine clinical care rather than obtained through a research study. However, errors in measurement (for example the limitation of the MMSE in comparison with more in-depth measures) will have obscured rather than exaggerated associations of interest. Considering sample representativeness, under the UK National Health Service structure, SLAM provides all mental health care (including dementia services) to its geographic catchment. Because there was minimal AChEI prescription by general practitioners during the period these data were collected, the sample represents all people in the catchment receiving these agents. However, people who receive AChEI intervention are only a subset of cases with dementia in the community. Perceived likelihood of a given outcome might have had an influence on inclusion, although this is unlikely because there are no commonly agreed factors predicting good or poor response (and indeed relatively few predictors of response were found in our analyses). Considering pre-treatment MMSE slopes, more precipitous decline might predict someone with dementia coming to medical attention and it is possible that this accounted at least in part for the slope improvement around AChEI initiation In terms of loss to follow-up, mortality rates were relatively low (26.8% by the end of the observation period). Although analyses were of all cognitive data during the observation period rather than restricted to those during AChEI treatment, withdrawal from treatment would render discharge and loss to follow-up more likely. Withdrawal from AChEIs is also an issue for the primary analyses. In long-term studies of AChEI, high rates of withdrawal from treatment have been reported [23]. This causes difficulties in interpreting the results. The reasons for dropout are complex and may include side effects, deterioration, co-morbidity, but also a wish to switch to other drugs as they become available. In the study by Wallin [23], the one-year completion rate was 66%, while in other studies it has ranged from 33 to 92% [24], [25]. In our study, 38% of patients received AChEIs for the entire 36 month period after initiation. Among the remaining 62% of patients, the mean (SD) AChEI exposure duration was 14 (9) months.

Another limitation in this study was related to statistical concepts. We used models that assumed that the MMSE has linear scaling properties, which is an assumption that has frequently been argued. One study [26] found curvilinear scaling properties for the MMSE and other common global cognitive tests. The shape for that curve was very similar to a curve published by Proust-Lima and colleagues using a very different methodology [27]. However, we did not use this methodology because we did not have item-level MMSE data available.

In summary, in this naturalistic observational study, changes in cognitive trajectories around AChEI initiation were interestingly similar to those reported in randomised controlled trials. Clearly the effect of these agents on cognitive decline in dementia has to be concluded from the trial evidence. However, sample sizes in trials are too small, even when combined, to provide data on predictors of good/poor response to treatments. Addressing these questions relies on large observational datasets and our findings suggest that the growing use of electronic health records, coupled with the ability to extract automated data from text fields on routine measures such as the MMSE, provides important potential opportunities for advancing research into routine interventions and their real-world outcomes.
